# Leukocyte Imbalances in Mucopolysaccharidoses Patients

**DOI:** 10.3390/biomedicines11061699

**Published:** 2023-06-13

**Authors:** Nuno Lopes, Maria L. Maia, Cátia S. Pereira, Inês Mondragão-Rodrigues, Esmeralda Martins, Rosa Ribeiro, Ana Gaspar, Patrício Aguiar, Paula Garcia, Maria Teresa Cardoso, Esmeralda Rodrigues, Elisa Leão-Teles, Roberto Giugliani, Maria F. Coutinho, Sandra Alves, M. Fátima Macedo

**Affiliations:** 1 Instituto de Biologia Molecular e Celular (IBMC), Universidade do Porto, 4200-135 Porto, Portugalc.piriquito@gmail.com (C.S.P.); imondragao@ua.pt (I.M.-R.); 2 Cell Activation & Gene Expression (CAGE), Instituto de Investigação e Inovação em Saúde (i3S), Universidade do Porto, 4200-135 Porto, Portugal; 3 Departamento de Ciências Médicas, Universidade de Aveiro, Campus Universitário de Santiago, Agra do Crasto, Edifício 30, 3810-193 Aveiro, Portugal; 4 Centro de Referência de Doenças Hereditárias do Metabolismo (DHM), Centro Hospitalar Universitário de Santo António, 4099-001 Porto, Portugalrocrff@gmail.com (R.R.); 5 Centro de Referência de Doenças Hereditárias do Metabolismo (DHM), Centro Hospitalar e Universitário Lisboa Norte (CHULN), 1649-035 Lisbon, Portugalpatricio.aguiar@campus.ul.pt (P.A.); 6 Faculdade de Medicina da Universidade de Lisboa, Universidade de Lisboa, 1649-190 Lisbon, Portugal; 7 Centro de Referência de Doenças Hereditárias do Metabolismo (DHM), Centro Hospitalar e Universitário de Coimbra, Centro de Desenvolvimento da Criança, 3000-075 Coimbra, Portugal; 8 Centro de Referência de Doenças Hereditárias do Metabolismo (DHM), Centro Hospitalar Universitário de São João (CHUSJ), 4200-319 Porto, Portugalesmeralda.rodrigues@hotmail.com (E.R.); e.leaoteles@gmail.com (E.L.-T.); 9 Hospital de Clínicas de Porto Alegre, Universidade Federal do Rio Grande do Sul, DASA e Casa dos Raros, Porto Alegre 90610-150, Brazil; rgiugliani@hcpa.edu.br; 10 Research and Development Unit, Department of Genetics, INSA, 4000-055 Porto, Portugal; francisca.coutinho@insa.min-saude.pt (M.F.C.); sandra.alves@insa.min-saude.pt (S.A.)

**Keywords:** lysosomal storage diseases, mucopolysaccharidoses, glycosaminoglycans, leukocytes, T cells, invariant natural killer T (iNKT) cells

## Abstract

Mucopolysaccharidoses (MPSs) are rare inherited lysosomal storage diseases (LSDs) caused by deficient activity in one of the enzymes responsible for glycosaminoglycans lysosomal degradation. MPS II is caused by pathogenic mutations in the *IDS* gene, leading to deficient activity of the enzyme iduronate-2-sulfatase, which causes dermatan and heparan sulfate storage in the lysosomes. In MPS VI, there is dermatan sulfate lysosomal accumulation due to pathogenic mutations in the *ARSB* gene, leading to arylsulfatase B deficiency. Alterations in the immune system of MPS mouse models have already been described, but data concerning MPSs patients is still scarce. Herein, we study different leukocyte populations in MPS II and VI disease patients. MPS VI, but not MPS II patients, have a decrease percentage of natural killer (NK) cells and monocytes when compared with controls. No alterations were identified in the percentage of T, invariant NKT, and B cells in both groups of MPS disease patients. However, we discovered alterations in the naïve versus memory status of both helper and cytotoxic T cells in MPS VI disease patients compared to control group. Indeed, MPS VI disease patients have a higher frequency of naïve T cells and, consequently, lower memory T cell frequency than control subjects. Altogether, these results reveal MPS VI disease-specific alterations in some leukocyte populations, suggesting that the type of substrate accumulated and/or enzyme deficiency in the lysosome may have a particular effect on the normal cellular composition of the immune system.

## 1. Introduction

Mucopolysaccharidoses (MPSs) are a subgroup of lysosomal storage diseases (LSDs) characterized by the progressive intra-lysosomal accumulation of non-degraded or partially degraded glycosaminoglycans (GAGs), which are also increased in urine, blood, and cerebrospinal fluid [1,2].

MPS II (MIM# 309900), or Hunter syndrome, is caused by pathogenic variants in the *IDS* gene and is transmitted in an X-linked recessive pattern, affecting almost exclusively males. So far, over 800 variants in the *IDS* gene have been reported as disease-causing mutations: 389 missense/nonsense, 75 splicing mutations, 70 small insertions, 168 small deletions, 20 small indels, 66 gross deletions, 6 gross insertions/duplications, and 22 complex rearrangements [3]. As a result of any of these mutations, the activity of the enzyme iduronate-2-sulfatase (EC 3.1.6.13) is either reduced or absent, which leads to storage of dermatan sulfate (DS) and heparan sulfate (HS) within the lysosomes [1,2,4]. MPS VI (MIM# 253200), or Maroteaux–Lamy syndrome, is an autosomal recessive inherited LSD that results from defects in the *ARSB* gene and causes the complete or partial loss of enzymatic activity of arylsulfatase B (or N-acetylgalactosamine-4-sulfatase; EC 3.1.6.12), leading to the accumulation of dermatan sulfate (DS) inside the lysosome [1,2,4,5]. The mutational spectrum of MPS VI includes over 200 variants known to cause the disorder, from missense/nonsense (188, the vast majority) to small deletions, insertions, or indels (28, 9 and 2, respectively). A few splicing mutations (16) and some major deletions (8) have also been reported. Unlike MPS II, however, up until now, no major insertions/duplications or complex rearrangements have been reported for MPS VI [3].

The prevalence of MPSs varies among ethnic groups, which will influence which type of MPS dominates a geographic area. When observing the prevalence of all MPS types in 33 countries, it was possible to conclude that Saudi Arabia provided the highest frequency, followed by Portugal, Brazil, the Netherlands, and Australia [2,6]. Briefly, Saudi Arabia showed a combined prevalence of 16.9 per 100,000 live births, a value that is probably owed to regional or consanguineous marriages, or even to founder effects. Portugal, on the other hand, has a combined birth prevalence of 4.8 per 100,000 live births, while the Netherlands and Australia have 4.5 and 4.46 per 100,000 live births, respectively [2,6]. In 2004, a study about the prevalence of LSDs in Portugal revealed that MPS II comprised 34% and MPS VI corresponded to 16% of all MPSs. In Saudi Arabia, MPS VI has the highest birth prevalence, comprising 46% of all the MPS cases diagnosed. Several other countries reported MPS II as the MPS with highest birth prevalence, as it is the example the Brazilian population, where it accounted for 37% of all MPS cases followed by MPS VI. Since the data from the Portuguese population presented high prevalence values of MPS II and VI, these two types of MPS were chosen as focus of our study [2,6,7]. It is, however, worth mentioning that even though there is a relatively uniform distribution of MPSs across the country, MPS I and II are actually exceptions to that pattern. According to the 2004 report, the former (MPS I) represented 13% of all MPS cases in the North and 35% in the rest of the country; in contrast, MPS II represented 34 and 8% of the MPS in the same respective regions. Therefore, depending on the region, the most prevalent form/type of MPS in Portugal may also MPS I [7]. Unfortunately, because of sample availability, MPS I could not be included in this study.

At the clinical level, MPS II and VI share many features, both being characterized by a chronic and progressive course, multisystem involvement, organomegaly, dysostosis multiplex, and coarse facial features. Hearing, vision, cardiovascular function, and joint mobility may be also affected. Both range from severe to attenuated forms that present different onsets, rates of progression, and degrees of clinical symptoms. There is, however, a fundamental difference between both disorders: in the severe forms of MPS II, cognitive decline is present, whereas in MPS VI, patients cognition is usually normal [1,2]. The MPS II attenuated form allows the individuals to reach adulthood, while with the severe form, patients have a life span of approximately two decades. In MPS VI, the slowly progressing forms generally present less pronounced or even atypical symptoms, which make them more difficult to diagnose. Therefore, the age of diagnosis can go from 9 to 42 years old, with an average of 23.5 years [8]. Enzyme replacement therapy (ERT) is available for both diseases. ERT is based on the infusion of a recombinant form of the defective enzyme that leads to a decrease in GAGs storage, and to a somatic improvement [1,9]. Despite their overall success, currently available ERT have some disadvantages, such as their high cost and the lifelong dependency on frequent infusions, which is caused by the limited half-life time of the recombinant enzymes. Furthermore, those infusions can elicit adverse reactions, ranging from rash, angioedema, and bronchoconstriction to anaphylaxis, all of which have already been reported in MPS II and MPS VI patients [10,11]. In fact, many patients develop antibodies to the recombinant enzyme (anti-ERT antibodies). Altogether, this means that the immune system may have an impact on ERT tolerability and on its long-term efficacy. That is why immunological responses to ERTs are still a topic that continues to be studied, as it has not yet been definitively clarified whether high-titer anti-ERT antibodies may, at least in some cases, reduce the treatment’s efficacy [12,13]. It is also worth mentioning that the currently approved therapies are not able to reach several tissues (including bones, cartilage, eye, heart valves and CNS) and to reduce, for instance, neuroinflammation [14], a process that is now known to play a key role in the overall MPS pathology. In fact, understanding the context, course, and duration of these neuroinflammatory responses has been the focus of numerous teams over the last years, who aim to understand and, ultimately, correct their corresponding physiological, biochemical, and behavioral consequences [15,16,17,18,19]. Nowadays, promising solutions are being actively studied; these include alternative administration routes for ERT, ERT with fusion proteins that bypass the blood brain-barrier, gene therapy, substrate reduction therapy, and anti-inflammatory and pharmacological read-through drugs [1,5,12,20,21].

MPS patients have recurrent upper respiratory infections, pneumonia, bronchitis, and middle ear infections, particularly in those affected with the severe forms [5,22,23]. However, it is not well documented if these episodes are caused by the generally poor health status of the severely affected patients, or by specific immune alterations. It is known that GAGs accumulation in MPSs is associated with inflammation, namely in the joints and in the central nervous system [15,24]. The existing body of research on MPSs suggests that GAG fragments may work as an endogenous danger-associated molecular pattern (DAMP), which would initiate an innate immune signaling pathway [5,25]. GAGs can be either exocytosed and bind to Toll-like receptor 4 (TLR4) or released intracellularly due to lysosomal disruption [5,17,20,26]. Indeed, GAGs are structurally similar to the canonical ligand for TLR4, which is lipopolysaccharide (LPS) [26]. Upon binding to TLR4, DAMP GAGs promote myeloid differentiation primary response (88 MyD88) adaptor protein recruitment, which, in turn, leads to the assembly of an interleukin receptor associated kinase (IRAK)4/IRAK1/IRAK2/TRAF6 complex. This complex then binds to a secondary one, TGF-β activated kinase (TAB) 2/TAK1/TAB1, allowing TRAF6 to dislocate from IRAK1 and move into the cytoplasm. In there, it can activate the NF-Kb pathway, which promotes pro-interleukin-1 β (pro-IL-1β) and NLR family pyrin domain containing 3 (NLRP3) transcription and transcriptional induction of other pro-inflammatory cytokines, culminating in the activation of NLRP3 inflammasome. Through cleavage of caspase-1, mature IL-1β will be secreted, together with the other pro-inflammatory cytokines and, all together, these will cause microglia and astrocyte activation, pyroptosis, and neuronal degeneration [20].

In addition to the alterations in innate immunity, the lysosomal alteration present in MPS can support the investigation of acquired immunity deficiencies, since the lysosome has a crucial role in antigen processing, autophagy, and perforin-induced lysis [17,20]. Several studies have revealed that MPS mouse models present irregularities in the function of the immune system. GAGs interact with a wide range of proteins, including chemokines, cytokines, growth factors, enzymes, and adhesion molecules, involved in physiological and pathological processes. Therefore, GAGs might induce inflammation in different cells [27]. Simonaro et al. examined articular chondrocytes from rats with MPS VI and showed an age-progressive increase in the number of apoptotic chondrocytes, also these cells released more nitric oxide (NO) and tumor necrosis factor alpha (TNF-α) when compared to control chondrocytes. It was suggested that apoptosis induction by accumulated GAG fragments might play an important role in cartilage destruction and degenerative joint disease in MPS VI [19]. Tessitore et al. studied the relationship between storage and secondary events, such as autophagy, polyubiquitination, mitochondrial function, inflammation, and apoptosis, in cells and tissues of MPS VI-affected rats. They detected abundant CD68-positive monocyte/macrophage cells, as well as apoptotic cells in liver, spleen, and kidney sections of affected MPS rats compared with controls [28]. In an MPS I mouse model, a decrease in circulating CD4^+^, CD8^+^ T cells, as well as dendritic cells (with decreased expression of cell surface CD123 and CD86) was reported [29]. A murine model for MPS VII showed reduced T cell proliferative response and decreased antibody production after immunization with antigens, possibly due to defective antigen processing [30]. In contrast, in a mouse model of MPS IIIB, there was increased T cell proliferation with mice showing increased numbers of splenic B cells as well as CD4^+^ and CD8^+^ T cells [17,31]. Concerning patients with MPS, much less data is available. A recent study reported significantly lower Ig levels, namely IgM and IgA, in MPS patients comparing with patients with other LSDs [32].

Thus, a wide range of alterations in the immune system were reported in different MPS diseases, and although a correlation between innate immunity and MPS has already been established, there is still insufficient data about how the adaptive immune system is affected in MPS patients. Given this, herein we performed a detailed characterization of lymphocyte populations in MPS II and MPS VI patients. More specifically, we analyzed the B, T, natural killer (NK) and invariant natural killer T (iNKT) cell populations.

## 2. Materials and Methods

### 2.1. Population Studied

This study was approved by the Ethical Committees of University Hospital Center São João, Porto, Portugal, that comprises São João Hospital and Valongo Hospital, University Hospital Santo António (Porto, Portugal), Lisbon Academic Medical Center—Santa Maria Hospital—North Lisbon University Hospital Center (Lisbon, Portugal), Pediatric Hospital—University Hospital of Coimbra (Coimbra, Portugal) and Hospital de Clínicas de Porto Alegre (Porto Alegre, Brazil). The informed consent was obtained from participants according to the Helsinki declaration.

### 2.2. Patient Population

Ten male MPS II disease patients (mean age: 15.4 ± 6.6 years; range 7–28 years) and sixteen MPS VI disease patients, comprising eight males and eight females (mean age: 15.4 ± 6.1 years; range 4–27 years), were included in this study. These patients were regularly followed up at São João Hospital (Porto, Portugal), Santo António Hospital (Porto, Portugal), Santa Maria Hospital (Lisbon, Portugal), Coimbra Pediatric Hospital (Coimbra, Portugal) and Hospital de Clínicas de Porto Alegre (Porto Alegre, Brazil). With the exception of two MPS II disease patients (both of them males with 12 and 17 years old), all individuals in both groups were already under ERT at the time of the analysis.

### 2.3. Control Population

Twenty-seven subjects who were apparently healthy were used as controls, including eighteen males and nine females (mean age: 16.8 ± 7 years; range 6–30 years). The control group was age matched with the patient group. The adult control group was composed of blood donors at the Blood Bank of São João Hospital (Porto, Portugal) and the child control group by children undergoing orthopedic surgery at Valongo Hospital (Porto, Portugal).

### 2.4. Isolation of Mononuclear Cells from Human Peripheral Blood and Flow Cytometry

Peripheral blood mononuclear cells (PBMCs) were isolated via Histopaque-1077^®^ (Sigma-Aldrich, St. Louis, MO, USA) density gradient centrifugation following the manufacturer’s instructions. Human PBMCs were stained with fluorochrome-conjugated antibody/tetramer mixes diluted in PBS/0.2%BSA/0.1%NaN_3_ (flow cytometry solution) for 20 min, at 4 °C, in the dark. To identify and phenotypically characterize the T cell, iNKT cell, NK cell, and B cell populations, the following monoclonal antibodies and tetramer were used: PerCP-Cy5.5-labeled anti-human CD3 (SK7) antibody (eBioscience San Diego, CA, USA); PE-Cy7-labeled anti-human CD4 (RPA-T4) antibody (eBioscience, San Diego, CA, USA); APC-eFluor^®^780-labeled anti-human CD8 (RPA-T8) antibody (eBioscience, San Diego, CA, USA); FITC-labeled anti-human CD19 (HIB19) antibody (eBioscience, San Diego, CA, USA); APC-labeled anti-human CD45RA (MEM-56) antibody (ImmunoTools, Friesoythe, Germany); eFluor^®^450-labeled anti-human CD161 (HP-3G10) antibody (ImmunoTools, Friesoythe, Germany); FITC-labeled anti-human CCR7 (150503) antibody (R&D Systems, Minneapolis, MN, USA); and PE-labeled PBS57-loaded hCD1d tetramer (National Institute of Health Tetramer Core Facility, Emory University, Atlanta, GA, USA). After staining, PBMCs were washed with flow cytometry solution and fixed with PBS 1% formaldehyde. In each flow cytometry experiment, the controls used were unstained sample; single stain samples for compensation; unloaded CD1d tetramer (to confirm the specificity of the CD1d PBS57 loaded tetramer staining); and healthy subject control. Sample acquisition was performed in a 3-laser BD FACSCanto™ II (BD Biosciences, San Diego, CA, USA) flow cytometer, using the BD FACSDiva™ software (BD Biosciences, San Diego, CA, USA). All flow cytometry analyses were performed using the FlowJo software (FlowJo LLC, Ashland, OR, USA).

### 2.5. Statistical Analysis

All statistical analyses were performed using the GraphPad Prism software v6 (GraphPad Software Inc., San Diego, CA, USA). The Shapiro–Wilk test was applied to determine the normal distribution of the variables. For the comparison of a variable between all groups (controls, MPS II, and MPS VI disease patients), the parametric one-way ANOVA test was applied when variables were normally distributed, while the non-parametric Kruskal–Wallis test was used for variables that did not follow a normal distribution. For the comparison between two groups (controls versus MPS II disease patients) t-student or Mann–Whitney tests were used. Values of *p* < 0.05 were considered statistically significant.

## 3. Results

### 3.1. Major Leukocyte Populations in MPS II and VI Disease Patients

We analyzed the peripheral blood mononuclear cells (PBMCs) of MPS II and MPS VI disease patients and compared with control subjects to infer whether GAG storage could interfere with immune cell composition.

**Figure 1:**
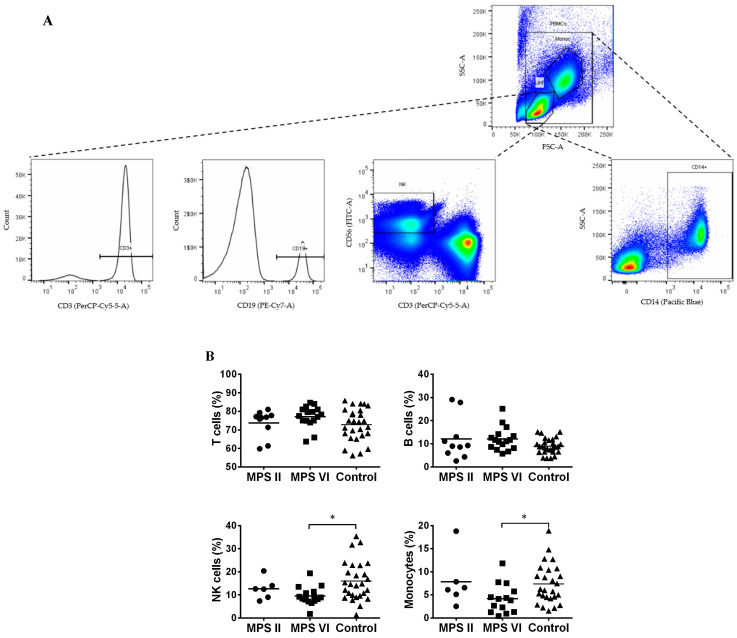
Major peripheral blood mononuclear cells (PBMCs) populations in MPS II, MPS VI disease patients, and control subjects. (**A**) Gating strategy used to identify T cells (CD3^+^); B cells (CD19^+^); NK cells (CD56^+^ CD3^−^) and monocytes (CD14^+^) populations. (**B**) Percentage of T cells, B cells and NK cells was determined among lymphocytes and of monocytes among PBMCs. Horizontal bars represent mean values. * *p* < 0.05.

PBMCs consist of monocytes, identified by the expression of CD14, and lymphocytes. Lymphocytes are composed of cells of innate immunity as NK cells, identified by CD56^+^CD3^−^ expression, and cells of the adaptive immunity, the B and T cells, identified by CD19 and CD3 expression, respectively. The gating strategy to identify these cells’ population is described in Figure 1A.

When comparing with control subjects, both the MPS II and MPS VI patient groups showed normal levels of T cells [33] and B cells (Figure 1B). However, we found a significant decrease in the percentage of NK cells (*p* = 0.0107) and monocytes (*p* = 0.0305) in MPS VI patients when compared with the control group (Figure 1B). Importantly, this alteration was absent in MPS II disease patients. This suggests that NK cells and monocytes seem to be affected in a disease-specific manner.

### 3.2. Naïve vs. Memory T Cell Pool in MPS II and VI Disease Patients

**Figure 2:**
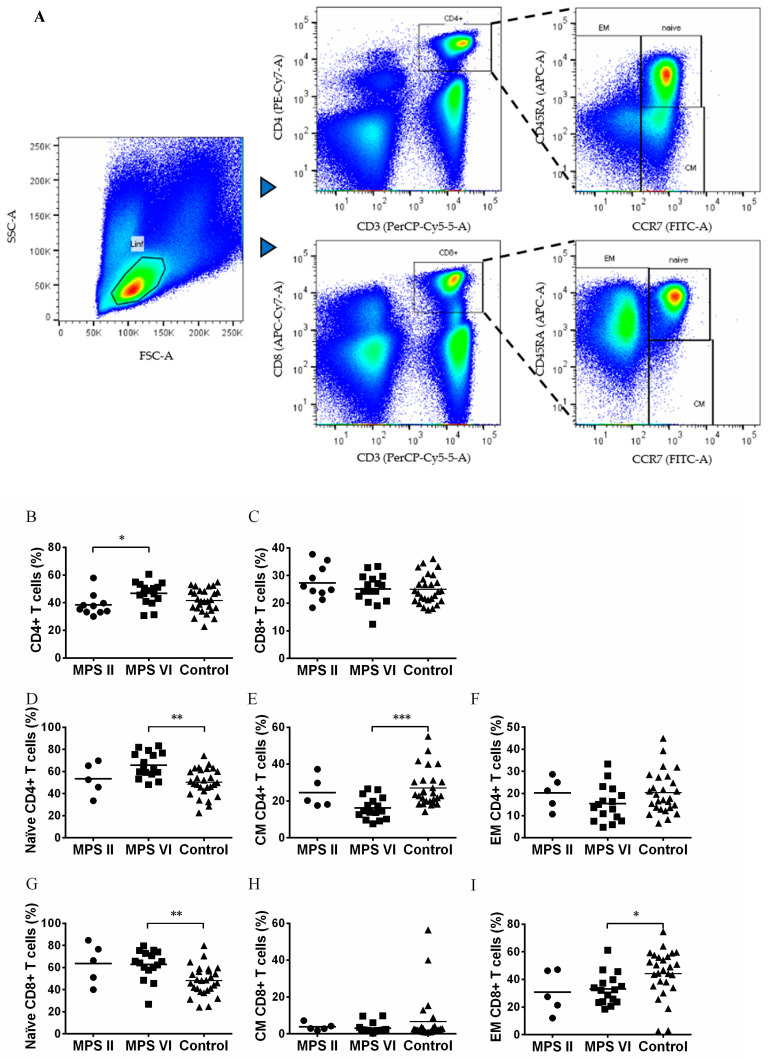
Frequency of naïve, central memory, effector memory CD4^+^ and CD8^+^ T cell subsets in MPS II, MPS VI disease patients, and control subjects. (**A**) Gating strategy applied selecting within the lymphocyte gate the populations of CD4^+^CD3^+^ (T helper cells) and CD8^+^CD3^+^ (cytotoxic T cells). Within each population: naïve, central memory (CM) and effector memory (EM) cell subset were detailed. (**B**) Percentage of CD4^+^ T cells in total lymphocytes, (**C**) Percentage of CD8^+^ T cells in total lymphocytes, (**D**) Percentage of naïve cells in CD4^+^ T cells, (**E**) Percentage of CM cells in CD4^+^ T cells, (**F**) Percentage of EM cells in CD4^+^ T cells, (**G**) Percentage of naïve cells in CD8^+^ T cells, (**H**) Percentage of CM cells in CD8^+^ T cells, and (**I**) Percentage of EM cells in CD8^+^ T cells. Horizontal bars represent mean values. * *p* < 0.05; ** *p* < 0.01; *** *p* < 0.001.

T lymphocytes are divided into different major functional groups: cytotoxic, identified by the expression of CD8, and helper cells, with a major immune modulatory role, identified by the expression of CD4 (Figure 2A). No alterations in the percentage of helper CD4^+^ and cytotoxic CD8^+^ T cells were observed between MPS II and MPS VI disease patients when compared to control subjects [33] (Figure 2B,C). Surprisingly, an increase in T helper cells was found in MPS VI disease patients (*p* = 0.0333) as compared with MPS II disease patients (Figure 2B).

The T helper and cytotoxic T cell populations were further characterized in terms of antigen experience into naïve T cells (CCR7^+^CD45RA^+^), central memory (CCR7^+^CD45RA^−^), and effector memory (CCR7^−^) (Figure 1A) in MPS II and MPS VI patients. In comparison with the control group, MPS VI patients presented a significant increase of naïve CD4^+^ T cells (*p* = 0.0029) and naïve CD8^+^ T cells (*p* = 0.0037), with a concomitant decrease in central memory CD4^+^ T cells (*p* = 0.0004) and effector memory CD8^+^ T cells (*p* = 0.03) (Figure 2D–I). Importantly, these differences cannot be attributed to age differences between MPS VI disease patients and control subjects, since they were age matched. On the contrary, MPS II disease patients did not show alterations in the memory state of both helper and cytotoxic T cells as compared with the control population, once again suggesting that these alterations are specific to MPS VI disease patients.

### 3.3. iNKT Cells Are Normal in Number but Phenotypically Altered in MPS II and MPS VI Disease Patients

**Figure 3:**
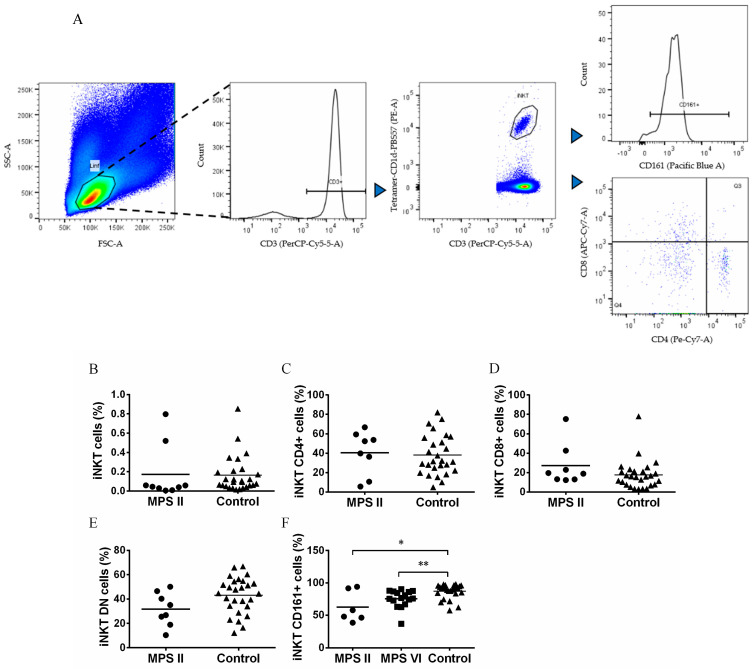
Percentage and phenotype of iNKT cells in MPS disease patients, and control subjects. (**A**) Gating strategy to characterize iNKT cells by the expression of CD4, CD8 and CD161 among total T cells. (**B**) Percentage of iNKT cells in total T cells of MPS II disease patients and controls, (**C**) CD4^+^CD8^−^ cells in iNKT cells of MPS II disease patients and controls, (**D**) CD4^−^CD8^+^ cells in iNKT cells of MPS II disease patients and controls, (**E**) CD8^−^/double negative (DN) cells in iNKT cells of MPS II disease patients and controls and (**F**) CD161^+^ cells in iNKT cells of MPS II and VI disease patients and control group. Horizontal bars represent mean values. * *p* < 0.05; ** *p* < 0.01.

Invariant natural killer T cells (iNKT) are lymphocytes acting in innate and adaptive immunity. They are identified by using CD1d loaded with αGalCer analogous (Figure 3A). iNKT cells are altered in several LSDs animal models and in acid sphingomyelinase deficiency patients [34,35,36,37]. Thus, we also analyzed the percentage of iNKT cells within the lymphocyte population via CD3 expression and reactivity to the PBS57 loaded CD1d tetramer, and iNKT cell phenotype via the expression of CD4, CD8 and CD161 (Figure 3A). We previous observed no alterations in the frequency and CD4/CD8 phenotype of iNKT cells in MPS VI disease patients [33]. Here, we extended this characterization to MPS II disease patients and went further in the analyses of iNKT cell phenotype by analyzing CD161 expression, an important NK cell marker. In MPS II disease patients, we found normal levels of iNKT cells among total T cells (Figure 3B) and a normal iNKT CD4/CD8/DN phenotype (Figure 3C–E). However, MPS II disease patients showed a decrease in the percentage of iNKT cells expressing the NK cell marker CD161 (*p* = 0.0364) as compared with control subjects (Figure 3F). Because of this result, we also investigated the expression of CD161 for MPS VI disease patients, and, in accordance with what was seen for MPS II disease patients, iNKT cells from MPS VI disease patients had a lower expression of CD161 when compared to control (*p* = 0.0087, Figure 3F).

## 4. Discussion

In the present study, we analyzed several immunological parameters in MPS II and MPS VI disease patients to investigate leukocyte modifications in order to understand if immune cells are altered in a disease-specific way and if they could play a role in the clinical course of the disease. We found a decrease in NK cells and monocytes in MPS VI disease patients, as well as alterations in the memory status of both cytotoxic and T helper cells in these patients. Importantly, these alterations were not present in MPS II disease patients. Additionally, we found a decrease in iNKT cells expressing the marker CD161 in both MPS VI and MPS II disease subjects.

A reduction in NK cells had already been described in patients with other LSDs such as Gaucher disease [38,39] and Niemann–Pick disease type C1 [40] and could potentially be due to a decrease in shingosine-1-phosphate (S1P) gradients and to defects in calcium storage. Monocytes were also decreased in Fabry disease patients’ blood, which could be related to an increase in extravasation processes with higher migration to peripheral tissues, since monocytes overexpress CD31, a molecule implied in extravasation processes [41]. The reduction in monocyte blood frequencies was also described in Gaucher disease. This reduction could be associated with the decrease in the CXCR4 expression (SDF1α receptor) that consequently leads to an inefficient CXCR4-SDF1α binding capacity and to a decline of monocyte SDF1α-dependent migration [42]. When compared to healthy children, children with Gaucher disease reported increases of activated T cells, T helper cells, and cytotoxic T cells [43,44]. These diseases share the characteristic of having sphingolipids as the main storage material. Here, we show for the first time that patients accumulating GAGs also develop defects in the frequency of these populations. The previous demonstration of disease-specific storage material in monocytes from MPS VI disease patients [45] led to the hypothesis that the accumulated GAGs might have a toxic effect in these cells, which may then cause a higher cellular apoptotic rate, thus explaining the decrease found. However, it is also possible that this reduction is mediated by an increased recruitment of monocytes to peripheral sites.

It is known that the lack or deficiencies in NK cells are associated with an increased susceptibility to pulmonary infections, a complication of MPS that often leads to death. This latter information was demonstrated in patients with total absence or functional abnormalities of NK cells [46,47]. This critical role of NK cells was also seen in a study with mice models of IL-15 KO deficient in NK cells and wild type mice depleted of NK cells, which were highly susceptible to pulmonary staphylococcal infection [48]. Consequently, our results may contribute to the explanation of the increased vulnerability to respiratory infections in MPS VI disease patients.

Interestingly, despite a normal percentage of total T cells, we show that MPS VI disease patients have an increase in the percentage of naïve helper and cytotoxic T cells, with concomitant decreases of their memory phenotypes, in comparison with the control group. Of note, these variations are not age-related, given that MPS VI disease patients and control subjects were age matched. These alterations were not present in MPS II disease patients. Interestingly, in an MPS I mouse model, which accumulates both dermatan and heparan sulfate (as MPS II disease patients), a reduction in peripheral blood memory CD4^+^ T cells was also found [29]. This indicates that, besides the nature of the accumulated material, the specific enzymatic defect may have an impact on immune cell homeostasis. A reduction in memory T cells might have important consequences for adaptive immune responses. The presence of a lower number of pathogen-specific T cells delays the response, increasing the risk of succumbing to infection. Thus, our results support the idea that the administration of vaccines against respiratory pathogens, to increase the pool of pathogen-specific memory T cells in MPS VI disease patients, might be beneficial [49]. Nevertheless, we cannot exclude the possibility that T cells may fail to expand, even after vaccination with a concomitant decrease in memory cells [50,51]. Indeed, patients with alpha-mannosidosis have a reduced response to vaccination when compared to healthy matched controls [50]. Furthermore, in mucolipidosis II (MLII) patients, the specific antibody response to vaccination is poor or not detectable, which is consistent with MLII mouse data. MLII patients have low levels of memory B cells, which indicates impaired B cell maturation in response to antigen, and defective immunoglobulin class switch, which prevents an effective response against pathogens [51].

Lysosomal integrity is important for iNKT cell development [36]. iNKT cells are immunomodulatory cells previously found to be decreased in several mouse models of LSDs [34,35,36,52,53,54] and in patients with acid sphingomyelinase deficiency [37]. In the present study, we found no differences in the frequency of iNKT cells between MPS II disease patients and control subjects, similar to what we had described for MPS VI disease patients [33]. However, we found a decreased expression of the maturation marker CD161, which is acquired through interactions with CD1d in the periphery [55] in both MPS II and MPS VI disease patients. The presence of alterations in both types of MPS suggests that this population might be particularly sensitive to lysosomal storage of GAGs.

A limitation of this study is the absence of information about patient’s genetic variants of *IDS* and *ARSB* genes, which would be important for correlation studies between the immunological findings and genetic variants.

All MPS VI disease patients and eight out of the total ten MPS II disease patients were under ERT at the time of the study. Therefore, we cannot exclude the possibility that some immunological alterations can be related to ERT. It would be important to include more untreated patients in this study to clarify this. Nevertheless, since most patients are under treatment, the clinical relevance of our findings persists.

## 5. Conclusions

In conclusion, with this study, we found a reduction in NK cells and monocytes in MPS VI disease patients, as well as alterations in the memory status of both cytotoxic and T helper cells in these patients. However, in MPS II disease patients, no alterations were seen in these cells. In addition, we detected a decrease in the expression of the marker CD161 in iNKT cells, both in MPS VI and MPS II disease patients.

These results lead to us believe that the type of substrate accumulated, or enzyme deficiency in the lysosome, may influence the normal cellular composition of the immune system, producing MPS VI disease-specific alterations in some leukocyte populations.

## Data Availability

The data presented in this study are available on request from the corresponding author. The data are not publicly available due to confidentiality of human subject data.
